# Sub-acute toxicity study of methanol extract of *Tetrorchidium didymostemon* leaves using biochemical analyses and gene expression in Wistar rats

**DOI:** 10.1016/j.heliyon.2020.e04313

**Published:** 2020-06-27

**Authors:** Osamudiamen Ebohon, Francis Irabor, Ehimwenma Sheena Omoregie

**Affiliations:** a Department of Biochemistry, Faculty of Natural and Applied Sciences, Michael and Cecilia Ibru University, Agbarha-Otor, Delta, Nigeria; b Malaria Research, Molecular Biology and Toxicology Unit, Department of Biochemistry, Faculty of Life Sciences, University of Benin, PMB 1154, Benin City, Nigeria

**Keywords:** *Tetrorchidium didymostemon*, Toxicity, Gene expression, Oxidative stress, Kidney function, Liver function, Subacute, Biological sciences, Biochemistry, Molecular biology, Toxicology, Pathophysiology

## Abstract

*Tetrorchidium didymostemon* is widely used by traditional medicine practitioners to manage and treat several diseases. Despite its known ethnomedicinal uses, there are no scientific studies on the toxic effects of this plant. This study was performed to evaluate the potential toxicity of methanol extracts *Tetrorchidium didymostemon* leaves through sub-acute oral administration in rats. Twenty four (24) male albino rats (Wistar strain) of average weight 150 ± 20 g were randomly divided into 4 groups of 6 rats each. Group 1 was the control while groups 2, 3 and 4 were administered 100, 300 and 600 mg/kg body weight of the plant extracts respectively for 14 consecutively days. The extract did not induce any treatment related changes in body weight, organ/body weight ratio, biochemical parameters (aspartate transaminase, alanine transaminase, total protein, albumin, creatinine and urea), oxidative stress indices (malondialdehyde, superoxide dismutase and reduced glutathione) and histopathology (liver and kidney) of the treated groups when compared to the control. However, at 600 mg/kg body weight dose, the extract caused a significant (*p* < 0.05) decrease in hemoglobin level, packed cell volume and the expression of albumin gene of rats. Similarly, at 300 and 600 mg/kg body weight, the extract also caused a non-significant (*p* > 0.05) decrease in red blood cell count. Furthermore, the extract at 100 and 300 mg/kg body weight induced a significant (*p* < 0.05) increase in the expression of tumor necrosis factor - alpha and kidney injury molecule - 1 (KIM-1) genes. Catalase gene expression especially in the kidney was up-regulated in the groups administered the extract. Our study suggests that oral administration of *T. didymostemon* leaves extract is relatively safe. However, there is need for caution due to the observed changes in hematological profile, up-regulation of KIM-1 genes as well as down regulation of albumin gene.

## 1 Introduction

Medicinal plants are used worldwide for the treatment of several diseases, and new drugs are continued to be developed via researches on these plants (Gupta et al., 2008). Medicinal plants contain wide array of bioactive compounds which are responsible for their therapeutic properties utilized in the treatment and management of several diseases. Bioactive compounds isolated from medicinal plants sometimes serve as a template on which novel synthetic drugs are designed. The use of medicinal plants to treat diseases without scientific validation on their safety and efficacy can be potentially dangerous (Mir et al., 2013). More worrisome is the fact that in developing countries, these medicinal plants are used without a scientifically proven effective dose and limit to the duration of usage. Furthermore, some medicinal plants contain compounds that are toxic and may pose serious harmful effects in man when consumed especially in high doses. Hence, it is pertinent to use validated scientific toxicity studies to establish medicinal plant safety (Bhushan et al., 2014).

*Tetrorchidium didymostemon* (Baill.) Pax & K. Hoffm is an evergreen shrub belonging to the Euphorbiaceae family, has drooping branches and can grow up to 25 m tall (Burkill, 2004). *Tetrorchidium* has about 25 species, of which 5 occurs in tropical Africa and the others in tropical America (Toirambe, 2008). The Edo people of Nigeria call it ‘iheni’, in Yoruba, it is called ‘ofun oke’ and in neighboring Cameroun it is called ‘efobolo’. In Edo, the leaves and stem bark of *T. didymostemon* are used for several medicinal purposes. The leaves are applied hot as a poultice to treat rheumatism or yaws; they are also used to dress enlarged limbs (Burkill, 2004). To treat enlarged spleen and constipation in babies, the leaves sap is applied to the nipples of nursing mother (Burkill, 2004). Leaves sap of *T. didymostemon* is applied to wounds as a hemostatic, the leaves in water or rum, or a stem bark decoction, is commonly taken to treat fever and as a purgative (Toirambe, 2008). The bark is febrifuge, diuretic, antidote, emetic, purgative and parasiticide. Bark scrapings are applied as an enema to treat malaria and backache, while the stem bark infusion is used to treat oedema, the latex from the stem are used as eye drops to treat filariasis, and it's applied to leprous sores, abscesses and grandular swelling (Toirambe, 2008).

Despite the wide usage of *T. didymostemon* in managing and treating several diseases*,* there is no scientific information on its safety when consumed orally. In other to fill in the gap, this study was aimed at evaluating the safety and toxicity of methanol extract of *T. didymostemon* leaves after 14 days consecutive daily oral administration in male Wistar rats.

## 2 Materials and methods

### 2.1 Plant collection and authentication

Leaves of *Tetrorchidium didymostemon* (Baill.) Pax & K. Hoffm were collected from the wild in Urhokuosa village (6.452980, 5.802755) in Uhumwonde Local Government Area of Edo State, Nigeria in March 2019. The plant was authenticated at the Department of Plant Biology and Biotechnology, University of Benin, Nigeria by Dr. H.A Akinnibosun and voucher specimen of the plant, UBH_T_439 was deposited at the herbarium of same department. The plant scientific name was checked with http://www.theplantlist.org on 11^th^ February, 2020.

### 2.2 Extract preparation

Fresh *T. didymostemon* leaves collected from the wild were air-dried and macerated. Extraction was carried out according to the method described by Al-Afifi et al. (2018). Three hundred grams (300 g) of the macerated leaves was soaked in 3 L of methanol (99.8% purity) at room temperature in air tight containers for 72 h and stirred occasionally. The resulting suspension was filtered using Whatman No 1 filter paper into a clean flask and the filtrates were concentrated using a rotary evaporator (RE 300, Bibby Scientific, UK) under reduced pressure at 45 °C and final concentrate was obtained using silica gel. The extracts were thereafter stored in a sterile container and kept in a refrigerator maintained at 4 °C. Methanol was used for extraction because it has high polarity and it's more effective at extracting polar phytochemicals such as flavonoids and phenolics (Anwar et al., 2010). Similarly, a review conducted by Tiwari et al. (2011) identified methanol as being able to extract more phytochemicals in comparison to water, ethanol, chloroform, ether and acetone.

### 2.3 Experimental animals

Male albino rats (Wistar strain) weighing between 150 – 170 g, bred in the Department of Anatomy, University of Benin, Benin City, Nigeria were used for the study. The rats were housed under standard laboratory conditions of light/dark cycle for 12 h at temperature 27 ± 2 °C with free access to standard commercial chow and water *ad-libitum*. The rats were allowed to acclimatize for two weeks before the experiment commenced. The research was conducted in accordance with the internationally accepted principles for laboratory animal use and care as found in the European Community guidelines (EEC Directive of 1986; 86/609/EEC). Ethical approval for this study was granted by the Institutional Ethics Review Committee, University of Benin (No: LS19114).

### 2.4 Subacute toxicity study

This study was designed with slight modification based on earlier procedure of Sathya et al. (2011). Twenty four (24) male rats of average weight 150 ± 20 g were randomly divided into 4 groups of 6 rats each. Group 1 rats served as the control and were administered the vehicle (0.7% carboxymethyl cellulose), once daily, orally for 14 days, consecutively. Group 2–4 animals were administered orally 100, 300 and 600 mg/kg body weight of methanol extract of *T. didymostemon* leaves respectively once daily for 14 consecutive days. The vehicle and extract were administered to the rats using oral gavage.

### 2.5 Sample collection

All animals were sacrificed by cervical dislocation 24 h after the last exposure to methanol extract of *T. didymostemon* leaves. They were fasted for 12 h before sacrifice. Blood was collected via aortic puncture and allowed to clot for 1 h and then centrifuged at 2000 rpm for 10 min to obtain serum. EDTA bottle was used to collect blood samples for hematological parameters. Liver, kidney, heart, spleen and testis were harvested and weighed. A portion of the liver and kidney (1 g) was homogenized in 5 ml of ice-cold 0.1 M phosphate buffer, pH 7.4 using a Potter–Elvehjem homogenizer. Post-mitochondrial fractions were obtained by centrifuging the homogenates at 12,000 g for 15 min at 4 °C. The post-mitochondrial fraction and serum were then used for biochemical assays. A small portion of the liver and kidney were fixed in 10% formalin for histological examination and RNAlater solution for RNA extraction.

### 2.6 Estimation of oxidative stress indices

Lipid peroxidation was assessed in terms of malondialdehyde (MDA) formation following previously described procedure Buege and Aust (1978). Superoxide dismutase (SOD) activity was determined using the method of Misra and Fridovich (1972) and reduced glutathione (GSH) level was estimated using previously described protocol Beutler et al. (1963).

### 2.7 Estimation of hepatic and renal function indices

Spectrophotometric method with Randox test kits for determining alanine aminotransferase (ALT) (Reitman and Frankel, 1957), aspartate aminotransferase (AST) (Reitman and Frankel, 1957), albumin (Grant et al., 1987) and total protein (Tietz, 1995) were used to estimate liver function indices. Similarly, Randox test kit for urea (Fawcett and Scott, 1960) and creatinine (Bartels et al., 1972) were used to estimate kidney function. The manufacturer's protocols were strictly followed in all instances.

### 2.8 Assessment of hematological parameters

Hematological analysis was carried out on blood collected in EDTA tube using an automated hematological analyzer (Beckman Coulter JT series Hematological Analyser). Hematocrit (HCT), hemoglobin (HGB), red blood cells (RBC), white blood cells (WBC), platelet (PLT) counts etc. were estimated.

### 2.9 Gene expression

#### 2.9.1 Total RNA isolation

Total RNA was isolated from whole tissues (liver and kidney) following the method described by Omotuyi et al. (2018). Briefly, tissues were homogenized in cold (4 °C) TRI reagent (Zymo Research, USA, Cat: R2050-1-50, Lot: ZRC186885). Total RNA was partitioned in chloroform (BDH Analytical Chemicals, Poole, England Cat: 10076-6B) following centrifugation at 15,000 rpm/15 min (Abbott Laboratories, Model: 3531, Lake Bluff, Illinois, United States). RNA from the clear supernatant was precipitated using equal volume of isopropanol (Burgoyne Urbidges & Co, India, Cat: 67-63-0). RNA pellet was rinsed twice in 70% ethanol (70 ml absolute ethanol (BDH Analytical Chemicals, Poole, England Cat: 10107-7Y) in 30 ml of nuclease-free water (Inqaba Biotec, West Africa, Lot no: 0596C320, code: E476-500ML)). The pellets were air-dried for 5 min and dissolved in RNA buffer (1 mM sodium citrate, pH 6.4).

#### 2.9.2 cDNA conversion

Prior to cDNA conversion, total RNA quantity (concentration (μg/ml) = 40 ∗ A_*260*_) and quality (≥1.8) was assessed using the ratio of A_260_/A_230_ (A = absorbance) read using spectrophotometer (Jen-way UV-VIS spectrophotometer model 6305, UK). DNA contamination was removed from RNA following DNAse I treatment (NEB, Cat: M0303S) as specified by the manufacturer. 2 μl solution containing 100 ng DNA-free RNA was converted to cDNA using M-MuLV Reverse transcriptase Kit (NEB, Cat: M0253S) in 20 μl final volume (2 μl, N^9^ random primer mix; 2 μl, 10X M-MuLV buffer; 1 μl, M-MuLV RT (200 U/μl); 2 μl, 10 mM dNTP; 0.2 μl, RNase Inhibitor (40 U/μl) and 10.8 μl nuclease-free water). The reaction proceeded at room temperature O/N. Inactivation of M-MuLV Reverse transcriptase was performed at 65°C/20 min.

#### 2.9.3 PCR amplification and agarose gel electrophoresis

Primers targeting exon-exon junction was designed using Primer3 software (Ye et al., 2012). All oligonucleotides synthesis was done by Inqaba Biotec, Pretoria, South Africa. PCR amplification for the determination of genes whose primers are listed below (Table 1) were done using the following protocol: PCR amplification was performed in a total of 25 μl volume reaction mixture containing 2 μl cDNA (40 ng), 2 μl primer (100 pmol) 12.5 μl Ready Mix Taq PCR master mix (One Taq Quick-Load 2x, master mix, NEB, Cat: M0486S) and 8.5 μl nuclease-free water. Initial denaturation at 95 °C for 5 min was followed by 20 cycles of amplification (denaturation at 95 °C for 30 s, annealing (see TM values for each primer pair on Table 1) for 30 s and extension at 72 °C for 60 s) and ending with final extension at 72 °C for 10 min. In all experiments, negative controls were included where reaction mixture has no cDNA. The amplicons were resolved on 2.0 % agarose gel (Cleaver Scientific Limited: Lot: 14170811) in Tris (RGT reagent, china, Lot: 20170605)-Borate (JHD chemicals, China, Lot 20141117)-EDTA buffer (pH 8.4). In-gel amplicon bands images captured on camera were processed on Keynote platform. Gel density quantification was done using Image-J software (Rueden et al., 2017). Each point represent relative expression ((test gene band intensity/internal control band intensity)∗100) plotted using Numbers software (Mac OSX version).

**Table 1:** Primer sequences.

Genes	Accession number	Primer sequences	Tm °C	Amplicon Size (bp)
Albumin	NM_134326.2	Forward (5′-3′): CAGCCTTGCAACACTTGTCC Reverse (5′-3′): CTCTCGCTGAGCTGGTGAAA	60	102
TNF-α	NM_012675.3	Forward (5′-3′): CATCCGTTCTCTACCCAGCC Reverse (5′-3′): AATTCTGAGCCCGGAGTTGG	57	146
Catalase	NM_012520.2	Forward (5′-3′): TCACCTGAAGGACCCTGACA Reverse (5′-3′): TCCATCTGGAATCCCTCGGT	55	103
KIM-1	NM_173149.2	Forward (5′-3′): TGTCACCATGTGTGGCTGAAT Reverse (5′-3′): GGCCAGCCCTCTAATGGTAA	60	148
GAPDH	NM_008084	Forward (5′-3′): AGATCCACAACGGATACATT Reverse (5′-3′): TCCCTCAAGATTGTCAGCAA	52	309

KIM-1 = Kidney injury molecule-1, TNF-α = Tumor necrosis factor alpha, GAPDH = Glyceraldehyde-3-phosphate dehydrogenase.

### 2.10 Histology

Thin sections of the liver and kidney fixed in 10% neutral buffered formalin were dissected and processed using Leica TP2010 automatic tissue processor for 18 h. The processor passed the tissues through fixation, dehydration, dealcoholisation, and paraffinization. Ultra-thin sections of 5 μm were sliced from the paraffinated sections using a Thermo scientific semi-automated rotary microtome. The tissues were then subjected to hematoxylin and eosin staining and viewed under a microscope using 10 X magnification.

### 2.11 Statistical analysis

The statistical analysis was performed using the statistical package for social science (SPSS) for windows, version 16.0 (SPSS Inc., Chicago, IL, USA). The results obtained were expressed as Mean ± SEM. One way analysis of variance (ANOVA) test was used to determine significance differences between the groups and post hoc multiple comparison test was done using Tukey's HSD (honest significant difference). Statistical significance was declared when P value was less than 0.05.

## 3 Results

### 3.1 Effect of methanol extract of *T. didymostemon* leaves on organ/Body weight ratio

The effect of 14 days oral administration of methanol extracts of *T. didymostemon* leaves on organ/body weight ratio of rats is shown in Table 2. The extract, at different doses, did not induce any significant (*p* > 0.05) changes in the organ/body weight ratio of the liver, kidney, heart, spleen and testis of the rats when compared to the control group.

**Table 2:** Effects of methanol extract of *T. didymostemon* leaves on organ/body weight ratio.

Dose	Liver	Kidney	Heart	Spleen	Testis
Control	0.033 ± 0.001^a^	0.007 ± 0.001^a^	0.004 ± 0.000^a^	0.003 ± 0.000^a^	0.012 ± 0.000^a^
100 mg/kg b. wt.	0.033 ± 0.002^a^	0.006 ± 0.000^a^	0.003 ± 0.000^a^	0.003 ± 0.000^a^	0.012 ± 0.001^a^
300 mg/kg b. wt.	0.033 ± 0.002^a^	0.006 ± 0.000^a^	0.003 ± 0.000^a^	0.003 ± 0.000^a^	0.010 ± 0.000^a^
600 mg/kg b. wt.	0.032 ± 0.001^a^	0.006 ± 0.000^a^	0.003 ± 0.000^a^	0.003 ± 0.000^a^	0.010 ± 0.000^a^

Values are mean ± SEM, n = 6 rats/group. Values bearing the same superscript are not significantly different, while those bearing different superscript are significantly different (*p* < 0.05). b. wt. = body weight.

### 3.2 Effect of methanol extract of *T. didymostemon* leaves on mean body weight

The effect of methanol extracts of *T. didymostemon* leaves on the mean body weight of rats following 14-day administration is shown in Table 3. The extract, at various doses (100, 300 and 600 mg/kg), did not elicit any observable changes in the body weight of the rats throughout the 14 days of the study in comparison with the control.

**Table 3:** Effect of methanol extract of *T. didymostemon* leaves on mean body weight.

Dose	Day 0	Day 7	Day 14
Control	167.71 ± 13.27	188.20 ± 12.41	198.00 ± 7.34
100 mg/kg b. wt.	160.17 ± 8.27	186.29 ± 6.01	196.58 ± 6.01
300 mg/kg b. wt.	164.71 ± 8.18	180.17 ± 6.58	190.17 ± 5.07
600 mg/kg b. wt.	162.00 ± 6.60	182.86 ± 6.76	194.33 ± 9.04

Values are mean ± SEM, n = 6 rats/group. b. wt. = body weight.

### 3.3 Effects of methanol extract of *T. didymostemon* leaves on oxidative stress indices of liver and kidney homogenates after 14-day exposure

The biochemical changes in oxidative stress indices (malondialdehyde, superoxide dismutase, and GSH) of the liver and kidney of rats after 14 days oral administration of methanol extract of *T. didymostemon* leaves is shown in Table 4. The plant extract did not show any significant (*p* > 0.05) effect on MDA level in the liver of rats when compared to the control. However, non-significant (*p* > 0.05) decrease in MDA level of the liver and significant (*p* < 0.05) decrease in MDA level of the kidney were observed in the rats at 600 mg/kg dose. No significant (*p* > 0.05) changes in SOD and GSH levels were recorded in all the groups administered the plant extract when compared to the control in this study. The groups of rats administered 300 and 600 mg/kg body weight of the plant extract showed non-significantly higher SOD activity in the liver and kidney in contrast to the control.

**Table 4:** Effects of *T. didymostemon* methanol leaves extract on oxidative stress indices of liver and kidney homogenates of rats.

Groups	MDA (moles MDA/mg tissue)	SOD (units/mg tissue)	GSH (μg/mL)
**Liver**			
Control	49.05 ± 6.84^a^	0.145 ± 0.003^a^	33.42 ± 1.77^a^
100 mg/kg b. wt.	49.70 ± 4.28^a^	0.151 ± 0.003^a^	33.12 ± 1.18^a^
300 mg/kg b. wt.	45.50 ± 3.68^a^	0.154 ± 0.004^a^	34.67 ± 2.00^a^
600 mg/kg b. wt.	30.57 ± 4.73^a^	0.154 ± 0.002^a^	33.62 ± 1.19^a^

**Kidney**			

Control	127.73 ± 3.67^a^	0.153 ± 0.005^a^	40.5 ± 1.74^ab^
100 mg/kg b. wt.	128.93 ± 2.62^a^	0.150 ± 0.003^a^	36.11 ± 0.98^a^
300 mg/kg b. wt.	121.13 ± 0.96^ab^	0.156 ± 0.002^a^	43.26 ± 2.11^b^
600 mg/kg b. wt.	115.74 ± 3.79^b^	0.156 ± 0.002^a^	40.68 ± 1.21^ab^

Values are mean ± SEM, n = 6 rats/group. Values bearing the same superscript are not significantly different, while those bearing different superscript are significantly different (*p* < 0.05). MDA = malondialdehyde, SOD = superoxide dismutase, GSH = reduced glutathione. b. wt. = body weight.

### 3.4 Effects of methanol extracts of *T. didymostemon* leaves on serum hepatic enzymes/Proteins after 14-day exposure

The effect of methanol extract of *T. didymostemon* leaves on serum hepatic enzymes (AST and ALT) and proteins (total protein and albumin) of rats after 14 days oral administration is shown in Table 5. There were no significant (*p* > 0.05) increase in markers for hepatic injury or stress in rats after administration of the plant extracts. The extract caused non-significant (*p* > 0.05) increase in albumin and total protein level in rats when compared to the control.

**Table 5:** Effects of methanol extracts of *T. didymostemon* leaves on serum hepatic enzymes and proteins in rats.

Dose	Total Protein (g/dL)	Albumin (g/dL)	AST (U/L)	ALT (U/L)
Control	6.88 ± 0.20^a^	3.07 ± 0.17^a^	97.05 ± 7.43^a^	44.21 ± 0.65^a^
100 mg/kg b. wt.	7.16 ± 0.21^a^	3.88 ± 0.21^a^	91.04 ± 2.89^a^	39.02 ± 3.02^a^
300 mg/kg b. wt.	7.18 ± 0.17^a^	3.88 ± 0.08^a^	102.97 ± 5.75^a^	43.69 ± 1.35^a^
600 mg/kg b. wt.	6.91 ± 0.09^a^	3.74 ± 0.26^a^	82.15 ± 3.78^a^	40.55 ± 4.12^a^

Values are mean ± SEM, n = 6 rats/group. Values bearing the same superscript are not significantly different, while those bearing different superscript are significantly different (p < 0.05). ALT = alanine aminotransferase, AST = aspartate aminotransferase. b. wt. = body weight.

### 3.5 Effects of methanol extracts of *T. didymostemon* leaves on serum creatinine and urea levels in rats

The effect of methanol extract of *T. didymostemon* leaves on renal function (creatinine and urea level) is shown in Table 6. Amongst the groups, there were no significant (*p* > 0.05) increase in creatinine and urea levels recorded in rats following oral administration of methanol extracts of *T. didymostemon* leaves.

**Table 6:** Effects of *T. didymostemon* methanol leaves extract on renal function indices in rats.

Dose	Creatinine (mg/dL)	Urea (mg/dL)
Control	1.44 ± 0.05^a^	31.39 ± 1.64^a^
100 mg/kg b. wt.	1.34 ± 0.02^a^	29.69 ± 1.69^a^
300 mg/kg b. wt.	1.33 ± 0.02^a^	32.28 ± 1.34^a^
600 mg/kg b. wt.	1.38 ± 0.06^a^	28.33 ± 2.10^a^

Values are mean ± SEM, n = 6 rats/group. Values bearing the same superscript are not significantly different, while those bearing different superscript are significantly different (p < 0.05). b. wt. = body weight.

### 3.6 Effects of methanol extract of *T. didymostemon* leaves on hematological indices of rats following 14-day exposure

The effect of methanol extract of *T. didymostemon* leaves on hematological indices (WBC, RBC, HGB, HCT, PLT etc) of rats following 14-day administration is shown in Table 7. The result for hematology showed that the extract did not induce significant (*p* > 0.05) changes in most of the hematological parameters evaluated when compared to the control. However, at 600 mg/kg dose the extract caused a significant (*p* < 0.05) decrease in HGB and PCV levels when compared to the control. Similarly, at 300 mg/kg dose the extract also caused a significant (*p <* 0.05) decrease in PCV. Furthermore, the extract caused a non-significant (*p >* 0.05) decrease in RBC at 300 and 600 mg/kg doses when compared to the control.

**Table 7:** Effects of methanol extract of *T. didymostemon* on hematological indices of rats.

Item	Control	100 mg/kg b. wt.	300 mg/kg b. wt.	600 mg/kg b. wt.
WBC (×10³/μl)	3.97 ± 0.53^a^	4.50 ± 0.30^a^	3.33 ± 0.37^a^	3.40 ± 0.00^a^
LYM (%)	84.23 ± 0.47^a^	82.73 ± 2.07^a^	80.50 ± 2.40^a^	72.93 ± 4.67^a^
MON (%)	4.83 ± 0.47^a^	8.30 ± 0.80^a^	7.27 ± 0.53^a^	7.97 ± 1.77^a^
GRAN (%)	10.93 ± 0.93^a^	8.97 ± 2.87^a^	12.23 ± 1.87^a^	19.10 ± 2.90^a^
LYM (×10³/μl)	3.33 ± 0.47^a^	3.77 ± 0.33^a^	2.67 ± 0.23^a^	1.87 ± 0.73^a^
MON (×10³/μl)	0.17 ± 0.03^a^	0.37 ± 0.03^b^	0.27 ± 0.03^ab^	0.17 ± 0.03^a^
GRAN (×10³/μl)	0.47 ± 0.03^a^	0.37 ± 0.07^a^	0.40 ± 0.10^a^	0.47 ± 0.13^a^
RBC (×10⁶/μl)	6.79 ± 0.40^ab^	7.12 ± 0.31^a^	4.92 ± 0.61^b^	5.07 ± 0.53^ab^
HGB (g/dL)	14.93 ± 1.23^a^	15.70 ± 0.60^a^	12.67 ± 1.37^ab^	9.09 ± 1.05^b^
PCV (%)	44.67 ± 2.97^a^	46.70 ± 1.50^a^	33.37 ± 0.23^b^	33.37 ± 1.76^b^
MCV (FL)	64.47 ± 1.17^a^	65.77 ± 0.77^a^	63.53 ± 2.63^a^	63.37 ± 0.91^a^
MCH (pg)	21.90 ± 0.50^a^	22.03 ± 0.13^a^	21.37 ± 0.97^a^	21.10 ± 0.30^a^
MCHC (g/dL)	33.30 ± 0.50^a^	33.57 ± 0.23^a^	33.73 ± 0.13^a^	31.63 ± 0.86^a^
RDW-SD (FL)	45.57 ± 1.43^a^	45.60 ± 0.70^a^	47.03 ± 6.43^a^	46.03 ± 8.42^a^
RDW-Cv (%)	18.87 ± 0.73^a^	18.87 ± 0.07^a^	18.20 ± 0.40^a^	17.73 ± 0.46^a^
PLT (×10³/μl)	436.33 ± 7.67^a^	570.67 ± 53.33^a^	793.67 ± 450.67^a^	640.33 ± 94.72^a^
MPV (FL)	8.20 ± 0.20^ab^	7.53 ± 0.03^a^	8.97 ± 0.97^ab^	10.17 ± 0.58^b^
PDW (%)	9.97 ± 0.27^a^	9.63 ± 0.07^a^	10.77 ± 2.07^a^	10.73 ± 0.32^a^
PCT (%)	0.35 ± 0.00^a^	0.43 ± 0.04^a^	0.79 ± 0.52^a^	0.65 ± 0.12^a^
P.LCR (%)	8.93 ± 1.73^a^	5.27 ± 0.73^a^	10.53 ± 10.53^a^	21.60 ± 4.80^a^

Values are mean ± SEM, n = 6 rats/group. Values bearing the same superscript are not significantly different, while those bearing different superscript are significantly different (*p* < 0.05). b. wt. = body weight, HCT = Hematocrit, HGB = Hemoglobin, ALP = Alkaline Phosphatase, WBC = White Blood Cells, RBC = Red Blood Cells, LYM = Lymphocytes, MON = Monocytes, GRAN = Granulocytes, MCH = Mean Corpuscular Hemoglobin, RDW = Red Cell Distribution Width, PCT = Plateletcrit, PDW = Platelet Distribution Width, MCV = Mean Corpuscular Volume, MCH = Mean Corpuscular Hemoglobin Concentration, PLT = Platelets, MPV = Mean Platelet Volume.

### 3.7 Effect of methanol extract of *T. didymostemon* leaves on the expression of Tumour necrosis factor - alpha (TNF - α), catalase and albumin genes in the liver of rats following 14 days oral administration

**Figure 1:**
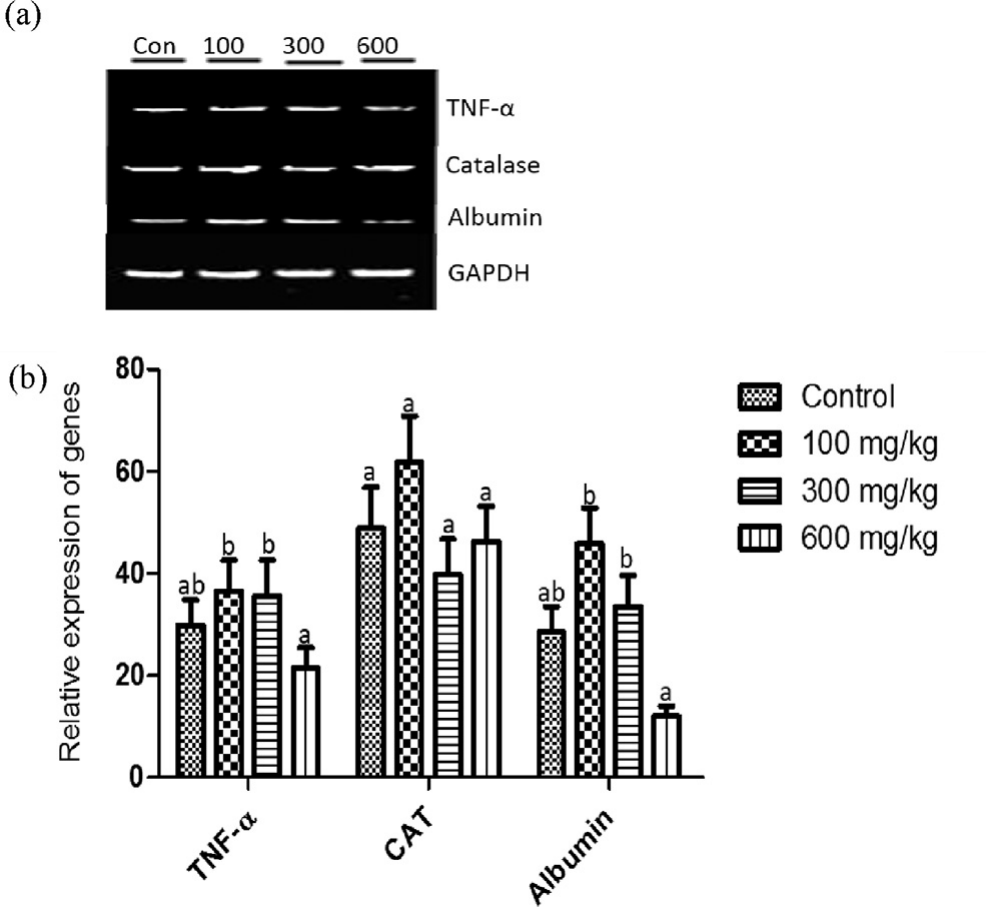
Effects of methanol extract of *T. didymostemon* leaves on the expression of tumor necrosis factor alpha (TNF-α), catalase and albumin genes in liver of rats following 14 days oral administration. Top panel (a) represent gel image showing mRNA expression of examined genes. Lower panel (b) represent densitometric analysis of TNF-α, catalase and albumin expression stated in top panel. Glyceraldehyde 3-phosphate dehydrogenase (GAPDH) was used as the loading control. Values are mean ± SEM, n = 6 rats/group. Columns bearing the same superscript are not significantly different, while those bearing different superscript are significantly different (*p* < 0.05). Con = control.

The result presented in Figure 1 shows the effect of 14-day oral administration of *T. didymostemon* leaves extract on the expression of TNF - α, catalase and albumin genes in the liver of rats. The rats administered the leaves extract at 100 and 300 mg/kg doses showed non-significant (*p* > 0.05) up-regulation in the expression of TNF – α gene in comparison to the control. However, at 600 mg/kg body weight, the extract down-regulated the expression of TNF – α gene in contrast to the control. Similarly, the administration of the extract, at 100 mg/kg body weight to rats, up-regulated, non-significantly (*p* > 0.05), the expression of catalase gene when compared to the control. At the other doses of the extracts, there were no significant (*p* > 0.05) changes in the expression of catalase gene. The expression of albumin gene was not significantly (*p* > 0.05) up-regulated in the groups of rats administered 100 and 300 mg/kg doses of the leaves extract while it was down-regulated in the 600 mg/kg group relative to the control values.

### 3.8 Effect of methanol extract of *T. didymostemon* leaves on the expression of tumor necrosis factor - alpha (TNF-α), catalase and kidney injury molecule - 1 (KIM-1) genes in the kidney of rats following 14 days oral administration

**Figure 2:**
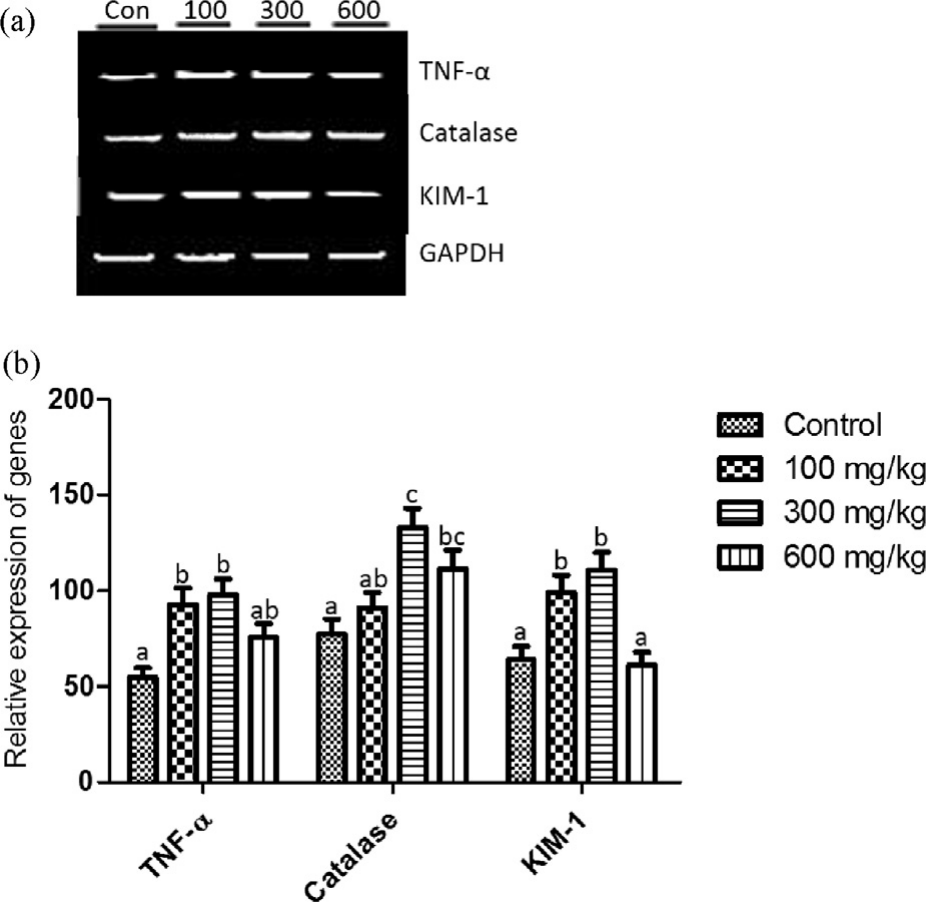
Effects of methanol extract of *T. didymostemon* leaves on the expression of tumor necrosis factor alpha (TNF-α), catalase and kidney injury molecule-1 (KIM-1) genes in kidney of rats following 14 days oral administration. Top panel (a) represent gel image showing mRNA expression of examined genes. Lower panel (b) represent densitometric analysis of TNF-α, catalase and albumin expression stated in top panel. Glyceraldehyde 3-phosphate dehydrogenase (GAPDH) was used as the loading control. Values are mean ± SEM, n = 6 rats/group. Columns bearing the same superscript are not significantly different, while those bearing different superscript are significantly different (*p* < 0.05). Con = control.

The result presented in Figure 2 shows the effect of 14-day oral administration of *T. didymostemon* leaves extract on TNF - α, catalase and KIM - 1 genes expression in the kidney of rats. The rats administered the leaves extract at 100 and 300 mg/kg doses exhibited significant (*p* < 0.05) up-regulation of TNF - α gene in comparison to the control rats. However, at 600 mg/kg body weight, the up-regulation of TNF - α gene was not significantly (*p* > 0.05) different from the control. Catalase gene expression was significantly (*p* < 0.05) up-regulated in the 300 and 600 mg/kg body weight groups relative to the control. Also, the expression of KIM - 1 gene was significantly (*p* < 0.05) up-regulated in the 100 and 300 mg/kg body weight extract treated groups compared to the control. However, KIM - 1 gene expression in rats given 600 mg/kg body weight of extract was not significantly (*p* > 0.05) different from that of the control.

### 3.9 Effect of methanol extract of *T. didymostemon* leaves on the histopathology of the liver and kidney of rats following 14 days oral administration

**Figure 3:**
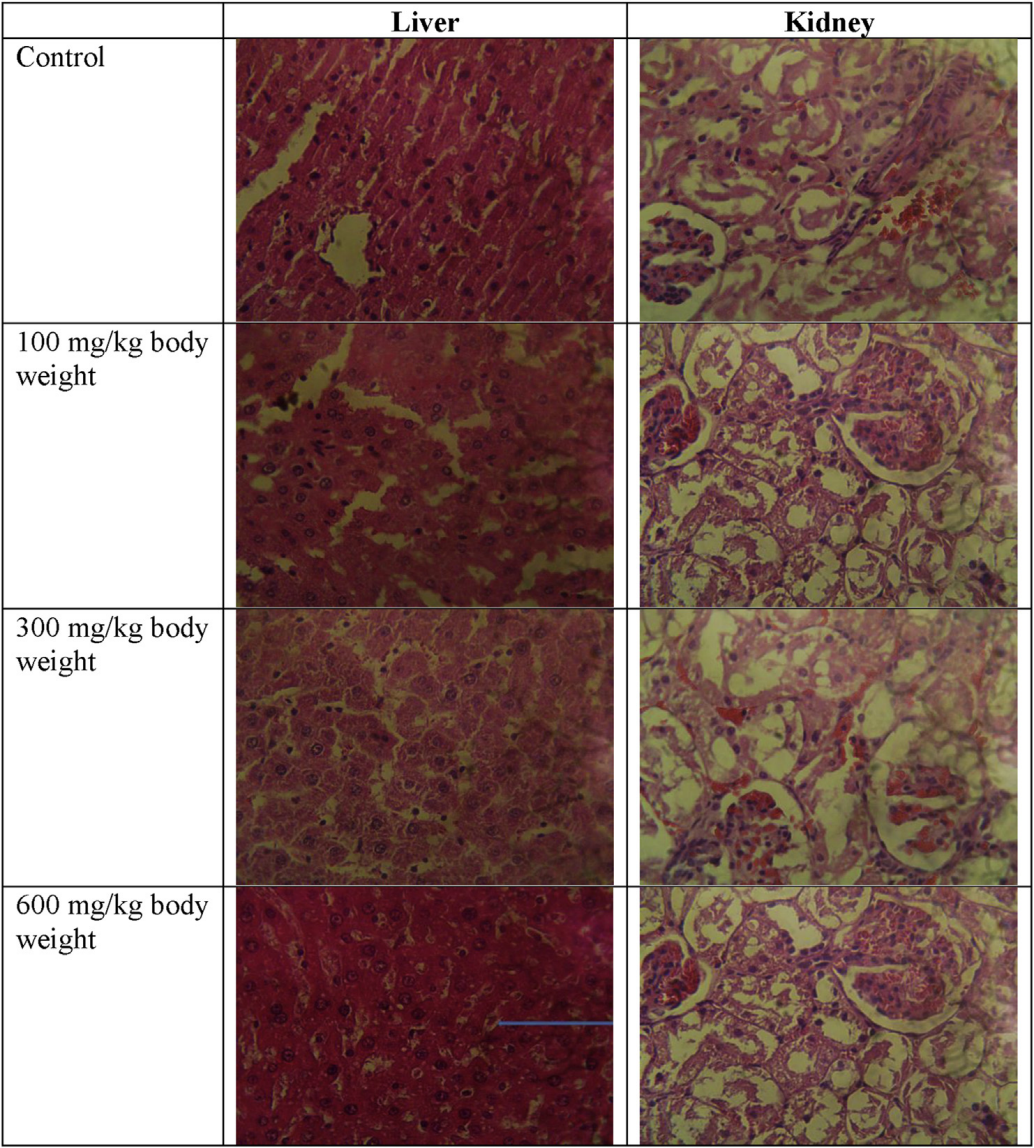
Photomicrograph of sections of liver and kidney in control and treated groups (H & E ×400). The liver section at 600 mg/kg shows mild degeneration of hepatocytes (thin arrow). Other sections of the liver were similar to the control. The sections of the kidney of the treated groups were similar to the control with normal glomerulus.

Histological examination of the liver and kidney sections of the extract administered groups of animals showed no changes when compared to the control. However, mild degeneration of the hepatocytes was observed in the rats administered the extract at 600 mg/kg dose. The kidney sections of the test groups were not also different from the control groups (Figure 3).

## 4 Discussion

Medicinal plants house several bioactive compounds with therapeutic potential for managing and treating ailments/diseases. Aside their health benefits, some of these compounds have been shown to have toxic effects. Hence, they are responsible for the toxicity associated with some medicinal plants. Herbal medicines are readily available in Africa; however, there are few scientific data to support the safety of these medicines. Sub-acute toxicity study is one of the ways to assess the safety and evaluate the effects of substances upon multiple exposures. This study is essential to establish a safe dose for human especially in developing nutraceuticals and pharmaceuticals. It is expected that the adverse effect of substance must happen within 14 days of administration in an ideal acute toxicity study (Bhardwaj and Gupta, 2012). In this study, there were no observed signs of toxicity such as death, tremors, writhing, convulsion, analgesia, increased motor activity, cyanosis and anesthesia in rats given the different doses (100, 300 and 600 mg/kg) of *T. didymostemon* leaves extract. The LD 50 of methanol leaves extract of *T. didymostemon* has been noted to be above 5000 mg/kg body weight (Ebohon et al*.,* Unpublished results).

Changes in the body weight as well as relative weight of organs gives an insight on the effects of an administered substance (Berenguer-Rivas et al., 2013). The result presented in this study shows that the extract did not cause any significant (*p* > 0.05) changes in the weight of all the organs studied as shown by the organ/body weight ratio. Similarly, the extract did not also cause alteration in the body weight of the animals when compared to the control. There was a gradual increase in body weight of rats in all the studied groups. This suggests that the extract may not have the potential to increase or support weight loss neither does it stimulate weight gain.

The liver and kidney are target organs for several substances due to their pertinent function in detoxification and excretion processes in the body (Bello et al., 2016). Hence, it is important for studies evaluating the biochemical effects of compounds to look deeply on the state of these organs. One mechanism of injury to these organs is via oxidative stress, which may result in the production of malondialdehyde (MDA) and 4-hydroxynonenal as well as alter antioxidant enzymes activities. In this study, the extract did not cause any significant (*p* > 0.05) change in hepatic MDA level when compared with the control. However, there was a non-significant (*p* > 0.05) decrease in hepatic MDA level in the groups of rats administered 300 and 600 mg/kg body weight. This implies that the extract at 300 and 600 mg/kg doses may have protective function against lipid peroxidation of the hepatocytes membrane, hence, the decrease in MDA level.

SOD protects cells from the deleterious effects of free radicals such as superoxide radicals by catalyzing the inactivation of superoxide anion to hydrogen peroxide. Again, in this study, there were no significant (*p* > 0.05) changes in hepatic SOD activity compared to the control group after oral administration of *T. didymostemon* leaves extract to rats for 14 days. We can infer that the extract did not stimulate the generation of superoxide radicals that could either reduce or induce SOD synthesis. This finding corroborates the previous study done by Badmus et al. (2014), where he recorded no changes in hepatic SOD activity after oral administration of *Garcinia kola* for 14 days. Similarly, no significant (*p* > 0.05) changes were seen in hepatic GSH level in the groups of rats administered *T. didymostemon* leaves extract compared to the control. GSH is used as a cofactor by several peroxidase enzymes to detoxify peroxides generated from oxygen radical metabolism (Strange et al., 2000). The lack of changes in hepatic GSH level may be an indication that the extract administration did not result in the generation of peroxides in the hepatocytes or it lacks the ability to induce GSH synthesis.

MDA (a product of lipid peroxidation) level of the kidney of the groups of rats administered *T. didymostemon* leaves extract was also not significantly (*p* > 0.05) different from that of the control group. Except for the group of rats that were given 600 mg/kg dose of the leaves extract that showed significant (*p* < 0.05) decrease in renal MDA level. This decrease may be attributed to the presence of antioxidants in the extract which might have scavenged free radicals responsible for lipid peroxidation. The trend in renal SOD activity was similar to that of hepatic SOD activity as there were no significant (*p* > 0.05) changes in the rats administered different doses of the extract compared to the control. Similarly, no significant (*p* > 0.05) changes were observed in the renal GSH level of the extracts treated groups compared to the control except the 300 mg/kg body weight group. The extract, through its bioactive constituents, as noted by Ebohon et al*.* (Unpublished results) may have modulated glutathione metabolism.

Enzymatic activities of the liver function enzymes (ALT, AST), total protein and albumin were used in this study to evaluate liver dysfunction. Serum activities of AST and ALT as well as the amount of total protein and albumin in the group of rats administered *T. didymostemon* leaves extract were not significantly (*p* > 0.05) different when compared to the control. Some serum enzymes such as AST and ALT have been noted to increase in cytotoxic and cholestatic hepatic injury (Zimmerman, 1978). ALT is found mainly in the liver and it's the most sensitive marker for hepatic cellular injury. Elevation of AST has been associated with hepatocellular injury in rats, while higher ALT activity has been linked to necrotic state (Navarro and Senior, 2006). Also, reduction of albumin in serum may suggest continued loss of albumin or infection (Yakubu et al., 2003). Changes in the volume of water and concentration of specific proteins in the plasma can result in changes in serum total protein (Loha et al., 2019). Thus, the non-significant changes in these hepatic injury markers suggest that *T. didymostemon* leaves extract did not result in hepatocellular injury, necrosis, disrupt cell membrane and inhibition of the secretory function of the liver. This observation is supported by the absence of histopathological changes in the liver except in the 600 mg/kg body weight group which had mild degeneration of the hepatocytes (Figure 3).

Measurement of urea, creatinine and electrolytes are used as indicators to assess renal dysfunction. However, creatinine test is a more specific marker to assess renal dysfunction than blood urea nitrogen. This is because exercise and high protein diet can influence the amount of urea in the blood. Plasma urea is usually increased in acute and chronic renal diseases while plasma creatinine is maintained mostly by glomerular filtration (Loha et al., 2019). In this study, the mean amount of creatinine and urea in the blood of the groups of rats administered the different doses (100, 300 and 600 mg/kg) of *T. didymostemon* leaves extract was not significantly (*p* > 0.05) different from the control group. This implies that the extract at these doses did not have any toxic effect on renal cells.

The hematopoietic system is one of the most sensitive targets of toxic compounds and is an important index of physiological and pathological status in human and animals (Adeneye et al., 2006). The plant extract did not show any significant effect on most of the hematological parameters studied in comparison to the control. However, it is important to note that at 300 and 600 mg/kg doses, the extract caused a significant (*p* < 0.05) decrease in packed cell volume (PCV) in rats when compared to the control. Similarly, the extract at 600 mg/kg body weight also causes a significant (*p* < 0.05) decrease in hemoglobin concentration in rats when compared to the control. Hence, there is need for caution when using *T. didymostemon* leaves extract as it has the potential of inducing anaemia either by interfering with hemoglobin concentration through inhibition of its synthesis or direct destruction of the protein. The presence of saponins in the methanol extract of *T. didymostemon* leaves as noted by Ebohon et al*.* (unpublished data) may be responsible for this effect. This is because saponins have been shown to have strong hemolytic effect and hence, disrupt erythrocytes.

In diseased tissues, genes are either up - or down - regulated and therefore, serve as a potential target for the development of drugs (Rastogi et al., 2015). Cytokines are key elements of the innate and adaptive immune systems; they provide a window through which diseases can be monitored and eventually controlled (Keustermans et al., 2013). For instance, TNF - α is a pro-inflammatory cytokine, which is involved in the pathogenesis of several autoimmune diseases and also stimulates the secretion of other inflammatory cytokines (Feng et al., 2011). High level of TNF - α has been associated in several inflammatory conditions and diseases such as malaria, coronary heart diseases, diabetes etc. In this study, there was a non-significant (*p* > 0.05) up-regulation of liver TNF-α gene in the group of rats administered 100 and 300 mg/kg body doses of *T. didymostemon* leaves extract in comparison to the control rats. However, TNF-α gene was down-regulated in the group administered 600 mg/kg dose of the extracts when compared to the control group. This finding suggest that the phyto-constituents present in *T. didymostemon* leaves extract may have anti-inflammatory property that is linked to its ability to down-regulate pro-inflammatory genes such as TNF-α gene at 600 mg/kg dose. The plant extract may have inhibited the activation of inflammatory mediator such as NF-κB: which when activated can up-regulate TNF-α, IL-1 and IL-8 (Ziamajidi et al., 2017). Relatedly, the inhibition of TNF-α expression may be as a result of the compounds from the plant extract interfering with signaling cascade leading to the expression of TNF-α or the induction of IL-4 known to inhibit TNF-α (Standiford et al., 1990). The overall effects of the extract may be linked to the presence of phyto-constituents such as flavonoids as noted by Ebohon et al. (Unpublished results). Flavonoids have been shown to modulate the expression of inflammatory genes and also the function of enzymes involved in inflammation (Middleton et al., 2000; Kim et al., 2004).

This study revealed non-significant (*p* > 0.05) changes in the expression of liver catalase gene of the groups of rats administered *T. didymostemon* leaves extract in comparison to the control. However, there was an up-regulation of catalase gene in the rats administered 100 mg/kg dose of the plant extract. Albumin gene was up-regulated in the groups of rats administered 100 and 300 mg/kg doses of the extract; but this up-regulation was not significantly different from that of the control values. This implies that the extract at lower doses may have the ability to stimulate albumin synthesis. We however recorded a non-significant down-regulation in expression of albumin gene in rats administered 600 mg/kg dose of *T. didymostemon* leaves extract compared to the control. Hence, there is need for caution in the use of *T. didymostemon* leaves extract at higher doses as it has the potential of interfering with the expression of albumin gene and may consequently result in decreased albumin synthesis as well as concentration. The short duration of this study may account for the reason why the effect of the extract on albumin gene expression at 600 mg/kg body weight dose did not result in significant decrease in serum albumin level.

In this study, the observed significant (*p* < 0.05) up-regulation in the expression of renal TNF-α gene in the group of rats administered 100 and 300 mg/kg and non-significant (*p* > 0.05) up-regulation in rats given 600 mg/kg doses of extract, suggests that, in the kidney, the extract may induce the synthesis of pro-inflammatory cytokines, in this case, TNF-α. Specific compounds in the extract probably stimulated the signal induced degradation of inhibitor of kappa B (IκB) protein and subsequently resulted in the activation of NF-κB needed for up-regulation of TNF-α. The extract induced an up-regulation of expression of renal catalase gene and this up-regulation was significantly higher in the 300 and 600 mg/kg body weight groups when compared to the control. *T. didymostemon* has been noted to have antioxidant potential (Ebohon et al*.,* unpublished data) and antioxidants can up-regulate the expression of genes coding for CAT, SOD, glutathione peroxidase and glutathione reductase (Aruoma, 1999; Kamalakkannan et al., 2005). These antioxidants such as phenolic acids can up-regulate transcription factor [nuclear factor erythroid 2-related factor 2 (Nrf2)] that modulates gene expression associated with antioxidant defense (Yeh and Yen, 2006). The plant extract may act by inducing processes that disrupt Keap-1 (Nrf2 inhibitor)/Nrf2 complex such as increased phosphorylation of Nrf2 serine or tyrosine residue. The activated Nrf2 is translocated to the nucleus where it binds to promoters containing antioxidant response elements, resulting in transactivation of genes required for antioxidant enzymes synthesis (Martín et al., 2010).

Due to the robust and marked expression of kidney injury molecule-1 (KIM-1) in an injured kidney, it has been suggested to be an ideal biomarker in several chemical and pathological nephrotoxicity models (Khandrika et al., 2012). In this study, KIM-1 gene was significantly up-regulated in the groups of rats administered 100 and 300 mg/kg doses of the extract in comparison to the control. However, KIM-1 gene was non-significantly down-regulated in the group administered 600 mg/kg dose of extract when compared to the control. The correlation between the TNF-α and KIM-1 gene expression at the 100 and 300 mg/kg body weight doses of extract suggest that *T. didymostemon* leaves extract may have the potential to induce slight dysfunction in the kidney associated with inflammation. However, this observation at the gene level did not result in significant changes in creatinine levels of the rats. Also, there were no observable histopathological changes in the kidneys of the extract administered animals. These findings agrees with the study conducted by Khandrika et al. (2012) who noted an up-regulation in KIM-1 mRNA and lack of changes in creatinine clearance in rats on ethylene glycol diet up to 3 weeks. It is possible that the up-regulation in KIM-1 gene expression in the group administered the extract was not significant enough to cause alteration in serum renal injury markers. Besides, the short duration of study may have prevented any changes in blood urea nitrogen and creatinine levels.

## 5 Conclusion

This study has demonstrated the safety of methanol extract of *T. didymostemon* leaves as its administration to rats at different doses did not result in lethality, alteration in serum markers for oxidative stress, hepatic and renal injury as well as histopathological changes. However, there is need for caution as it appears to be hemotoxic at higher doses (300 and 600 mg/kg) of the extract. In addition, the up-regulation of expression of renal TNF-α and KIM-1 genes observed in this study cannot be ignored. *T. didymostemon* methanol leaves extract may therefore be considered relatively safe, within the limit of the doses evaluated in this study, for therapeutic purposes. The authors recommend prolonged toxicity study on *T. didymostemon* leaves in other to fully validate its safety.

## Declarations

### Author contribution statement

O. Ebohon: Conceived and designed the experiments; Performed the experiments; Analyzed and interpreted the data; Contributed reagents, materials, analysis tools or data; Wrote the paper.

F. Irabor: Conceived and designed the experiments; Performed the experiments; Contributed reagents, materials, analysis tools or data.

E.S. Omoregie: Conceived and designed the experiments; Contributed reagents, materials, analysis tools or data.

### Funding statement

This research did not receive any specific grant from funding agencies in the public, commercial, or not-for-profit sectors.

### Competing interest statement

The authors declare no conflict of interest.

### Additional information

No additional information is available for this paper.
